# Association of body mass index and its classifications with gray matter volume in individuals with a wide range of body mass index group: A whole-brain magnetic resonance imaging study

**DOI:** 10.3389/fnhum.2022.926804

**Published:** 2022-09-08

**Authors:** Shinsuke Hidese, Miho Ota, Junko Matsuo, Ikki Ishida, Yuuki Yokota, Kotaro Hattori, Yukihito Yomogida, Hiroshi Kunugi

**Affiliations:** ^1^Department of Mental Disorder Research, National Institute of Neuroscience, National Center of Neurology and Psychiatry, Kodaira, Japan; ^2^Department of Psychiatry, Teikyo University School of Medicine, Itabashi-ku, Japan; ^3^Department of Neuropsychiatry, Division of Clinical Medicine, Faculty of Medicine, University of Tsukuba, Tsukuba, Japan; ^4^Department of Psychiatry, National Center Hospital, National Center of Neurology and Psychiatry, Kodaira, Japan; ^5^Department of Behavioral Medicine, National Institute of Mental Health, National Center of Neurology and Psychiatry, Kodaira, Japan; ^6^Medical Genome Center, National Center of Neurology and Psychiatry, Kodaira, Japan; ^7^Araya Inc., Minato-ku, Japan

**Keywords:** BMI, body mass index, gray matter volume (GMV), magnetic resonance imaging, obese, overweight, underweight

## Abstract

**Aim:**

To examine the association of body mass index (BMI) [kg/m^2^] and its classifications (underweight [BMI < 18.5], normal [18.5 ≤ BMI < 25], overweight [25 ≤ BMI < 30], and obese [BMI ≥ 30]) with brain structure in individuals with a wide range of BMI group.

**Materials and methods:**

The participants included 382 right-handed individuals (mean age: 46.9 ± 14.3 years, 142 men and 240 women). The intelligence quotient was assessed using the Japanese Adult Reading Test. Voxel-based morphometry (VBM) and diffusion tensor imaging (DTI) were performed to analyze the association of BMI and its classifications with gray and white matter structures, respectively.

**Results:**

According to VBM, BMI was significantly and negatively correlated with the bilateral cerebellum exterior volumes. In group comparisons, the right cerebellum exterior volume was significantly lower in the overweight or obese group than in the underweight or normal group, while the bilateral cuneus and calcarine cortex, left cuneus, and left precuneus volume was significantly lower in the underweight group than in the non-underweight group. Sex-related stratification analyses for VBM revealed that BMI was significantly and negatively correlated with the bilateral cerebellum exterior volumes only in women. In group comparisons, the left cerebellum exterior volume was significantly lower in obese women than in non-obese women. The left thalamus proper and the right cerebellum exterior volumes were significantly lower in overweight or obese group than in underweight or normal group in men and women, respectively. The bilateral cuneus and calcarine cortex, left cuneus and carcarine cortex, and bilateral cuneus volume was significantly lower in underweight men than in non-underweight men. In contrast, there were no notable findings on DTI.

**Conclusion:**

Our results suggest association of continuous BMI, being overweight or obese, and being underweight with decreased gray matter volume in individuals with a wide range of BMI group. Furthermore, sex-related differences are seen in the association of BMI and its classifications with regional gray matter volume reductions. Abnormally high or low BMIs may have a negative influence on regional gray matter volumes.

## Introduction

Magnetic resonance imaging (MRI) studies have suggested associations between body mass index (BMI) [kg/m^2^] and brain structure. Recent meta-analyses have reported that obesity is associated with lower regional gray matter volumes (Garcia-Garcia et al., 2019; Herrmann et al., 2019), while a review reported that BMI increase and obesity are negatively associated with regional white matter integrity (Kullmann et al., 2015). However, studies on the effects of BMI classifications (i.e., underweight, normal, overweight, and obese), especially being underweight or overweight, on brain structure have been still scarce, which warranted to investigate the association of not only BMI but also being underweight or overweight with gray and white matter structures.

BMI showed positive and/or negative correlations with regional gray matter volumes in 690 men (Taki et al., 2008), 109 individuals (Yao et al., 2016), two independent samples comprising 330 and 347 individuals (Opel et al., 2017), and 653 individuals (Huang et al., 2019). Obese individuals (BMI ≥ 30, *n* = 21) had smaller total gray matter volume than normal (18.5 ≤ BMI < 25, *n* = 117) and overweight (25 ≤ BMI < 30, *n* = 63) individuals (Gunstad et al., 2008), while obese men (BMI > 28, *n* = 20) showed increased putamen volume than lean men (18.5 ≤ BMI < 23.9, *n* = 20; Zhang et al., 2017).

BMI positively or negatively correlated with regional white matter fractional anisotropy (FA) and axial, radial, and mean diffusivity (MD) values in 51 (Xu et al., 2013) 33 (Kullmann et al., 2016) individuals, while BMI negatively correlated with widespread white matter FA values in two independent cohorts of 364 and 1064 (Repple et al., 2018) and in 1255 (Zhang R. et al., 2018) individuals. Obesity (BMI ≥ 30, *n* = 17) was associated with lower corpus callosum FA values in 103 individuals (Stanek et al., 2011). Obese individuals (BMI ≥ 30, *n* = 15) showed lower regional white matter axial diffusivity (AD) values than in lean (BMI < 25, *n* = 15) individuals (van Bloemendaal et al., 2016). Regional white matter AD, radial diffusivity (RD), and MD values were lower in obese (BMI ≥ 30, *n* = 20) individuals than in non-obese (*n* = 30) individuals, while BMI negatively correlated with regional white matter FA, AD, and RD values and positively correlated with focal white matter MD values in total 50 individuals (Chen et al., 2018).

Although various correlations between BMI and gray matter volume (Taki et al., 2008; Yao et al., 2016; Opel et al., 2017; Huang et al., 2019) or white matter microstructures (Xu et al., 2013; Kullmann et al., 2016; Chen et al., 2018; Repple et al., 2018; Zhang R. et al., 2018) have been reported, consistent results have not obtained. Similarly, inconsistencies have been included among effects of obesity on gray matter volume (Gunstad et al., 2008; Zhang et al., 2017) and white matter microstructures (Stanek et al., 2011; van Bloemendaal et al., 2016; Chen et al., 2018). However, these inconsistencies among previous relatively small sample studies are probably inevitable considering that reproducible brain-wide association studies require MRI samples with thousands of individuals (Marek et al., 2022).

Previous studies have thus suggested associations of body weight with gray and white matter structure; however, many of the previous studies are complicated and even contradictory. Besides, our 1.5 Tesla MRI study found no association between BMI or obesity (BMI ≥ 30) and gray matter volume and white matter integrity in 294 healthy individuals, while such an association was found in 307 patients with major depressive disorder (Hidese et al., 2018). Since these inconsistencies are considered to be reasonable from a recent MRI reproducibility study (Marek et al., 2022), we aimed to examine the association between being underweight (BMI < 18.5) or overweight (25 ≤ BMI < 30) and brain (gray and white matter) structures in 382 individuals with a wide range of BMI group which covered all its classifications of underweight, normal, overweight, and obese, because such examinations have not or rarely been performed previously.

## Materials and methods

### Participants

Participants included 382 right-handed Japanese individuals (mean age: 46.9 ± 14.3 years, 142 men and 240 women). The participants were recruited through the National Center of Neurology and Psychiatry (NCNP) hospital, an advertisement in a free local magazine, and an announcement on our laboratory website. The participants were included if they didn’t have any axis I psychiatric disorders screened using the Mini International Neuropsychiatric Interview (Sheehan et al., 1998; Otsubo et al., 2005) and individuals with a medical history of psychiatric disorders according to the Diagnostic and Statistical Manual of Mental Disorders 5th edition (American Psychiatric Association [APA], 2013), neurological diseases, severe head injury, substance abuse, and intellectual disability were excluded by certified psychiatrists. The study was explained, and written informed consent was obtained from all participants. The study protocol was approved by the Ethics Committee of the NCNP and was performed according to the latest version of the Declaration of Helsinki (World Medical Association, 2013).

### Clinical assessments

BMI [kg/m^2^] was classified according to the World Health Organization criteria (World Health Organization [WHO], 2000): underweight (BMI < 18.5), normal (18.5 ≤ BMI < 25), overweight (25 ≤ BMI < 30), and obese (BMI ≥ 30). Intelligence quotient (IQ) was assessed using the Japanese Adult Reading Test (JART; Matsuoka et al., 2006) face-to-face version, which was consisted of 100 Kanji compound words as described previously (Hidese et al., 2020).

### Statistical analyses

Comparisons of continuous variables [i.e., age, education level, BMI, JART score, and total intracranial volume (TICV)] were assessed by the analysis of variance (ANOVA) and the chi-square test for dichotomous variables, while comparisons of total gray matter, white matter, cerebrospinal fluid (CSF) volumes were assessed by the analysis of covariance (ANCOVA), controlling for age, sex, JART score, and TICV, between the underweight and non-underweight groups, between overweight or obese and underweight or normal groups, and between obese and non-obese groups. Correlations between BMI and clinical variables (i.e., age, education level, JART score, and TICV) were assessed using Pearson’s correlation coefficient (the Welch’s *t*-test was alternatively used for sex), while correlations of BMI with total gray matter, white matter, and CSF volumes were assessed using Pearson’s partial correlation coefficient, controlling for age, sex, JART score, and TICV. The effect sizes were evaluated using η^2^ for the ANCOVA and ANOVA and φ for the chi-square test. Statistical analyses were performed using the Statistical Package for the Social Sciences version 28.0 (SPSS Japan, Tokyo, Japan). All the statistical tests were two-tailed, and *p* < 0.05 was deemed significant.

### Magnetic resonance imaging data processing and analyses

High spatial resolution, three-dimensional T1-weighted, and diffusion tensor imaging (DTI) images were obtained using a 3.0 Tesla MR system (Trio, Siemens, Erlangen, Germany). Detailed information on MRI parameters has been described previously (Ota et al., 2017; Hidese et al., 2020). Cases were excluded if aberrant findings (e.g., arachnoid cyst, hemorrhage, infarction, and tumor) were detected in the raw MRI data. Preprocessing of T1-weighted images was performed using the Computational Anatomy Toolbox (CAT) version 12.8.1.^1^ Voxel-based morphometry (VBM) processing was performed using the CAT 12 running within statistical parametric mapping (SPM) version 12,^2^ in which default settings were used, and finally obtain modulated, spatially normalized gray matter images were further spatially smoothed with an 8-mm full-width at half-maximum Gaussian kernel. No threshold was not used in VBM. Anatomical regions were determined for Montreal Neurological Institute T1 atlas coordinates using “Neuromorphometrics” function in the SPM 12. Total intracranial volume (TICV) was calculated within the CAT 12 as the sum of the total gray matter, white matter, and CSF volumes. DTI data were processed using tract-based spatial statistics (Smith et al., 2006). The FA threshold was set to 0.20 to exclude peripheral tracts. The skeletonized FA, AD, RD, and MD values from the DTI were analyzed using the Functional MRI of the Brain Software Library “Threshold-Free Cluster Enhancement” option with 10,000 permutations in the “randomise” menu (Nichols and Holmes, 2002; Smith and Nichols, 2009).

The correlation of BMI with the gray matter volume and white matter FA, AD, RD, and MD values was assessed, controlling for age, sex, JART score, and TICV (only in VBM), and the correlation of JART score with the gray matter volume and white matter FA, AD, RD, and MD values was assessed, controlling for age, sex, and TICV (only in VBM). Differences in gray matter volume and white matter FA, AD, RD, and MD values were assessed between the underweight and non-underweight groups, between overweight or obese and underweight or normal groups, and between obese and non-obese groups, controlling for age, sex, JART score, and TICV (only in VBM). In the whole-brain analyses, statistical significance was set at a peak-level of *p* < 0.001 (uncorrected) and a cluster-level of *p* < 0.05 [false discovery rate (FDR) corrected] in VBM, and *p* < 0.05 (family-wise error corrected) in DTI.

## Results

**FIGURE 1:**
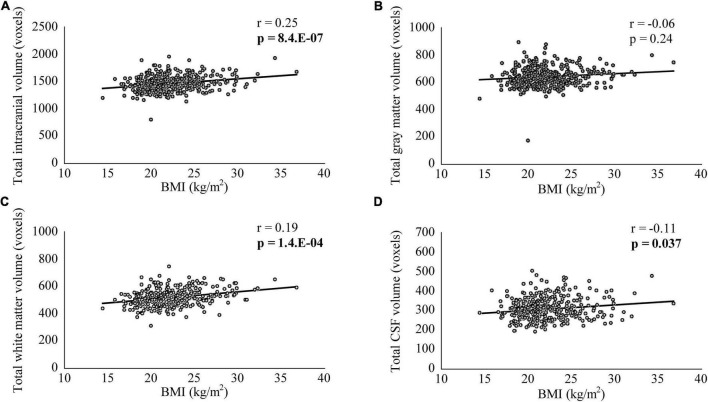
The correlation between body mass index (BMI) and total intracranial, gray matter, white matter, and cerebrospinal fluid (CSF) volumes. BMI was significantly and positively correlated with total intracranial **(A)** and white matter volumes **(C)**, while it was significantly and negatively correlated with total CSF volume **(D)**. There was no significant correlation between BMI and total gray matter volume **(B)**.

Characteristics and comparisons of clinical variables between the overweight or obese and underweight or normal groups are shown in Table 1. The proportion of men was significantly higher (*p* < 0.001), while the JART score was significantly lower (*p* < 0.01) in the overweight or obese group than in the underweight or normal group. Comparisons of variables between the underweight and non-underweight groups and between the obese and non-obese groups are shown in Tables 2, 3, respectively. The proportion of men was significantly lower in the underweight group than in the non-underweight group (*p* < 0.05), while the JART score was significantly higher in the obese group than in the non-obese group (*p* < 0.05). The correlations between BMI and the variables are shown in Table 4. BMI was significantly higher in men (mean: 23.3 ± 3.3 kg/m^2^) than in women (mean: 21.5 ± 2.9 kg/m^2^, *p* < 0.000001). BMI was significantly and positively correlated with TICV and total white matter volume (*p* < 0.001, Figures 1A,C), while it was significantly and negatively correlated with total CSF volume (*p* < 0.05, Figure 1D). There was no significant correlation between BMI and total gray matter volume (Figure 1B).

**TABLE 1:** Characteristics and comparisons of clinical variables between the overweight or obese and underweight or normal groups.

	Healthy individuals (*n* = 382)	Range	Overweight or obese group (*n* = 62)	Underweight or normal group (*n* = 320)	Statistical comparions
Age (years)	46.9 ± 14.3	18–75	48.1 ± 11.0	46.7 ± 14.8	*F* = 0.49, *p* = 0.48, η^2^ = 0.002
Men (%)	142 (37.2)		35 (56.5)	107 (33.4)	χ^2^ = 11.78, ***p* = 6.0.E-4**, φ = −0.176
Education (years)	14.8 ± 2.2	9–22	14.5 ± 2.0	14.8 ± 2.2	*F* = 0.94, *p* = 0.33, η^2^ = 0.003
BMI (kg/m^2^)	22.1 ± 3.2	14.4–36.8	27.6 ± 2.4	21.1 ± 2.0	*F* = 509.77, *p*** = 3.4.E-72**, η^2^ = 0.573
Obese (BMI ≥ 30) (%)	6 (1.6)		6 (9.7)	0 (0.0)	χ^2^ = 31.46, ***p* = 2.0.E-8**, φ = 0.287
Overweight (25 ≤ BMI < 30) (%)	56 (14.7)		56 (90.3)	0 (0.0)	χ^2^ = 338.68, ***p* = 1.2.E-75**, φ = 0.942
Normal (18.5 ≤ BMI < 25) (%)	290 (75.9)		0 (0.0)	290 (90.6)	χ^2^ = 233.30, ***p* = 1.1.E-52**, φ = −0.781
Underweight (BMI < 18.5) (%)	30 (7.8)		0 (0.0)	30 (9.4)	χ^2^ = 6.31, ***p* = 0.012**, φ = −0.129
Japanese Adult Reading Test	80.6 ± 11.4	46–99	76.6 ± 13.8	81.3 ± 10.7	*F* = 9.07, ***p* = 0.0028**, η^2^ = 0.024
Total intracranial volume (voxels)	1474.1 ± 142.6	805.8–1969.4	1525.9 ± 139.3	1464.1 ± 141.3	*F* = 10.00, ***p* = 0.0017**, η^2^ = 0.026
Total gray matter volume (voxels)	645.0 ± 69.8	174.3–896.7	658.5 ± 59.0	642.4 ± 71.5	*F* = 1.08, *p* = 0.30, η^2^ = 0.001
Total white matter volume (voxels)	520.3 ± 58.9	314.7–750.1	549.2 ± 61.6	514.7 ± 56.8	*F* = 10.68, ***p* = 0.0012**, η^2^ = 0.007
Total cerebrospinal fluid volume (voxels)	308.8 ± 56.9	194.0–506.4	318.2 ± 57.2	307.0 ± 56.7	*F* = 3.06, *p* = 0.08, η^2^ = 0.004

BMI, body mass index. Values are mean ± standard deviation. Significant p-values are shown in bold cases.

**TABLE 2:** Comparisons of variables between the underweight and non-underweight groups.

	Underweight group (*n* = 30)	Non-underweight group (*n* = 352)	Statistical comparions
Age (years)	46.7 ± 17.0	47.0 ± 14.0	*F* = 0.01, *p* = 0.93, η^2^ = 0.001
Men (%)	5 (16.7)	137 (38.9)	χ^2^ = 5.86, ***p* = 0.015**, φ = 0.124
Education (years)	15.1 ± 2.4	14.8 ± 2.1	*F* = 0.55, *p* = 0.46, η^2^ = 0.002
BMI (kg/m^2^)	17.7 ± 0.9	22.5 ± 3.0	*F* = 76.89, ***p* = 6.2.E-17**, η^2^ = 0.169
Obese (BMI ≥ 30) (%)	0 (0.0)	6 (1.7)	χ^2^ = 0.52, *p* = 0.47, φ = −0.037
Overweight (25 ≤ BMI < 30) (%)	0 (0.0)	56 (15.9)	χ^2^ = 5.59, ***p* = 0.018**, φ = −0.121
Normal (18.5 ≤ BMI < 25) (%)	0 (0.0)	290 (82.4)	χ^2^ = 102.63, ***p* = 4.1.E-24**, φ = −0.518
Underweight (BMI < 18.5) (%)	30 (100.0)	0 (0.0)	Not applicable
Japanese Adult Reading Test	83.3 ± 10.3	80.3 ± 11.5	*F* = 1.88, *p* = 0.17, η^2^ = 0.005
Total intracranial volume (voxels)	1401.4 ± 111.2	1480.3 ± 143.4	*F* = 8.63, ***p* = 0.0035**, η^2^ = 0.023
Total gray matter volume (voxels)	617.7 ± 55.0	647.4 ± 70.5	*F* = 0.04, *p* = 0.84, η^2^ = 0.001
Total white matter volume (voxels)	485.0 ± 46.4	523.3 ± 58.9	*F* = 3.43, *p* = 0.07, η^2^ = 0.003
Total cerebrospinal fluid volume (voxels)	298.8 ± 56.1	309.6 ± 56.9	*F* = 2.45, *p* = 0.12, η^2^ = 0.003

BMI, body mass index. Values are mean ± standard deviation. Significant p-values are shown in bold cases.

**TABLE 3:** Comparisons of variables between the obese and non-obese groups.

	Obese group (*n* = 6)	Non-obese group (*n* = 376)	Statistical comparions
Age (years)	40.7 ± 10.1	47.0 ± 14.3	*F* = 1.18, *p* = 0.28, η^2^ = 0.004
Men (%)	3 (50.0)	139 (37.0)	χ^2^ = 0.43, *p* = 0.51, φ = −0.034
Education (years)	15.7 ± 1.5	14.8 ± 2.2	*F* = 1.01, *p* = 0.32, η^2^ = 0003
BMI (kg/m^2^)	32.9 ± 2.3	22.0 ± 2.9	*F* = 86.59, ***p* = 1.1.E-18**, η^2^ = 0.186
Obese (BMI ≥ 30) (%)	6 (100.0)	0 (0.0)	Not applicable
Overweight (25 ≤ BMI < 30) (%)	0 (0.0)	56 (14.9)	χ^2^ = 1.05, *p* = 0.31, φ = −0.052
Normal (18.5 ≤ BMI < 25) (%)	0 (0.0)	290 (77.1)	χ^2^ = 19.22, ***p* = 1.2.E-5**, φ = −0.224
Underweight (BMI < 18.5) (%)	0 (0.0)	30 (8.0)	χ^2^ = 0.52, *p* = 0.47, φ = −0.037
Japanese Adult Reading Test	92.2 ± 7.6	80.4 ± 11.3	*F* = 6.42, ***p* = 0.012**, η^2^ = 0.017
Total intracranial volume (voxels)	1612.3 ± 190.3	1471.9 ± 141.0	*F* = 5.79, ***p* = 0.017**, η^2^ = 0.016
Total gray matter volume (voxels)	701.9 ± 60.4	644.1 ± 69.6	*F* = 0.73, *p* = 0.39, η^2^ = 0.001
Total white matter volume (voxels)	572.4 ± 57.8	519.4 ± 58.6	*F* = 0.07, *p* = 0.80, η^2^ = 0.001
Total cerebrospinal fluid volume (voxels)	338.0 ± 86.2	308.3 ± 56.3	*F* = 0.17, *p* = 0.68, η^2^ = 0.001

BMI, body mass index. Values are mean ± standard deviation. Significant p-values are shown in bold cases.

**TABLE 4:** The correlations between body mass index and the variables.

	Body mass index	
	*r* or *t*	*p*
Age (years)	0.09	0.09
Sex	5.33	**2.1.E-07**
Education (years)	−0.06	0.28
Japanese Adult Reading Test	−0.06	0.26
Total intracranial volume	0.25	**8.4.E-07**
Total gray matter volume	−0.06	0.24
Total white matter volume	0.19	**1.4.E-04**
Total cerebrospinal fluid volume	−0.11	**0.037**

*r*, Pearson’s or Pearson’s partial correlation coefficient. Significant *p*-value are shown in bold cases.

**FIGURE 2:**
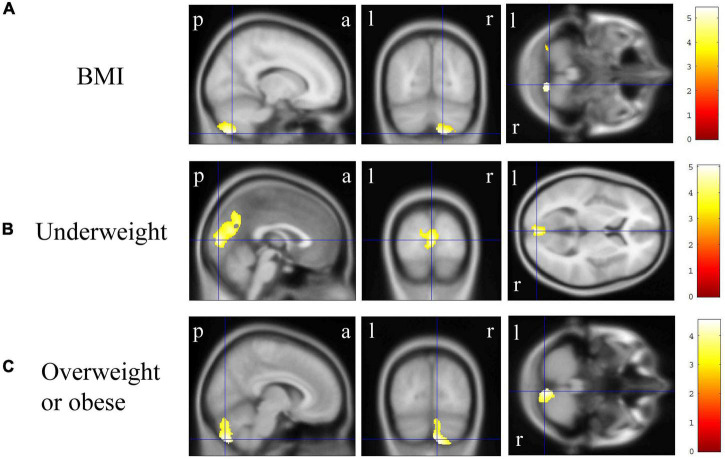
Gray matter regions where the volume was associated with body mass index (BMI), underweight, and overweight or obese individuals. **(A)** BMI was negatively correlated with gray matter volumes in the bilateral cerebellum exterior [*p* < 0.01 corrected, Montreal Neurological Institute (MNI) 152 T1 atlas coordinates: *x* = 15.0 mm, *y* = −70.5 mm, *z* = −58.5 mm indicated by blue cross-hair lines]. **(B)** Gray matter volume in the bilateral cuneus and calcarine cortex, left cuneus, and left precuneus was reduced in underweight individuals when compared with that in non-underweight individuals (*p* < 0.001 corrected, MNI 152 T1 atlas coordinates: *x* = 0.0 mm, *y* = −88.5 mm, *z* = 6.0 mm indicated by blue cross-hair lines). **(C)** Gray matter volume in the right cerebellum exterior was reduced in overweight or obese individuals when compared with that in underweight or normal individuals (*p* < 0.05 corrected, MNI 152 T1 atlas coordinates: *x* = 7.5 mm, *y* = −79.5 mm, *z* = −51.0 mm indicated by blue cross-hair lines). Right-sided gradient color bars represent *t*-scores. a, anterior; l, left; p, posterior; r, right.

**FIGURE 3:**
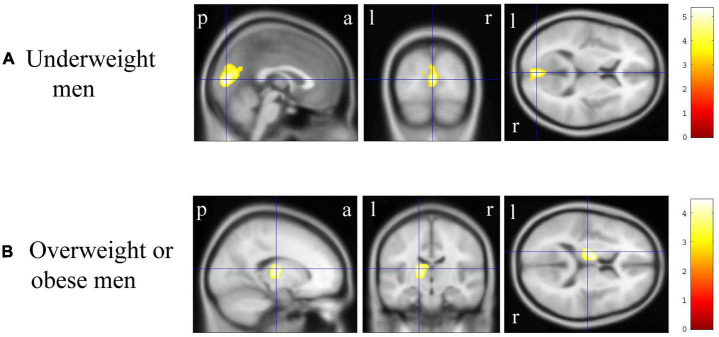
Gray matter regions where the volume was associated with underweight and overweight or obese men. **(A)** Gray matter volume in the bilateral cuneus and calcarine cortex, left cuneus and calcarine cortex, and bilateral cuneus was reduced in underweight men when compared with that in non-underweight men [*p* < 0.01 corrected, Montreal Neurological Institute (MNI) 152 T1 atlas coordinates: *x* = 0.0 mm, *y* = −84.0 mm, *z* = 9.0 mm indicated by blue cross-hair lines]. **(B)** Gray matter volume in the left cerebellum exterior was reduced in overweight or obese men when compared with that in underweight or normal men (*p* < 0.05 corrected, MNI 152 T1 atlas coordinates: *x* = −16.5 mm, *y* = −16.5 mm, *z* = 12.0 mm indicated by blue cross-hair lines). Right-sided gradient color bars represent *t*-scores. a, anterior; l, left; p, posterior; r, right.

**FIGURE 4:**
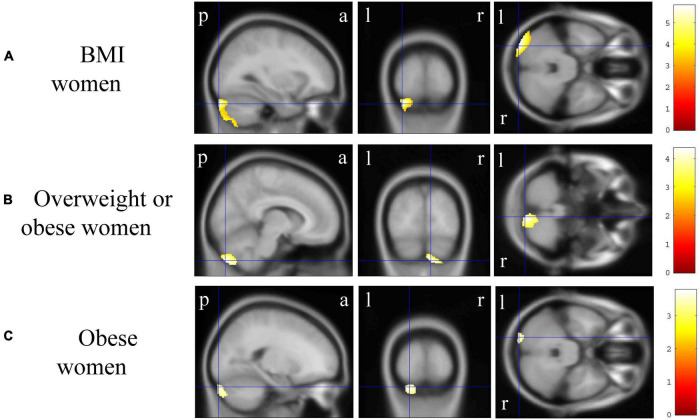
Gray matter regions where the volume was associated with body mass index (BMI), overweight or obese, and obese women. **(A)** BMI was negatively correlated with gray matter volumes in the bilateral cerebellum exterior [*p* < 0.01 corrected, Montreal Neurological Institute (MNI) 152 T1 atlas coordinates: *x* = −30.0 mm, *y* = −93.0 mm, *z* = −31.5 mm indicated by blue cross-hair lines]. **(B)** Gray matter volume in the right cerebellum exterior was reduced in overweight or obese women when compared with that in underweight or normal men (*p* < 0.05 corrected, MNI 152 T1 atlas coordinates: *x* = 9.0 mm, *y* = −84.0 mm, *z* = −51.0 mm indicated by blue cross-hair lines). **(C)** Gray matter volume in the left cerebellum exterior was reduced in obese women when compared with that in non-obese women (*p* < 0.01 corrected, MNI 152 T1 atlas coordinates: *x* = −21.0 mm, *y* = −94.5 mm, *z* = −30.0 mm indicated by blue cross-hair lines). Right-sided gradient color bars represent *t*-scores. a, anterior; l, left; p, posterior; r, right.

In VBM, BMI was significantly and negatively correlated with the bilateral cerebellum exterior volumes (Figure 2A, corrected *p* < 0.01). There was no significant positive correlation between BMI and regional gray matter volume. In group comparisons, the bilateral cuneus and calcarine cortex, left cuneus, and left precuneus volume was significantly lower in the underweight group than in the non-underweight group (Figure 2B, corrected *p* < 0.001). The right cerebellum exterior volume was significantly lower in the overweight or obese group than in the underweight or normal group (Figure 2C, corrected *p* < 0.05), while there was no significant gray matter volume reduction in the obese group when compared to the non-obese group. No gray matter regions were significantly higher in the underweight, overweight or obese, or obese groups. There was no significant correlation between JART score and regional gray matter volume. Based on the VBM results in total participants, sex-related stratification analyses were further performed using the similar correlational and group comparison methods, controlling for age, JART score, and TICV. BMI showed no significant correlation with regional gray matter volume in men. The bilateral cuneus and calcarine cortex, left cuneus and calcarine cortex, and bilateral cuneus volume was significantly lower in underweight men than in non-underweight men (corrected *p* < 0.01), while the left thalamus proper volume was significantly lower in overweight or obese men than in underweight or normal men (Figure 3, corrected < 0.05). BMI was significantly and negatively correlated with the bilateral cerebellum exterior volumes in women (Figure 4A, corrected *p* < 0.01). The right cerebellum exterior volume was significantly lower in overweight or obese women than in underweight or normal women (corrected *p* < 0.05), while the left cerebellum exterior volume was significantly lower in obese women than in non-obese women (Figures 4B,C, corrected *p* < 0.01). The VBM statistics are shown in Table 5.

**TABLE 5:** The voxel-based morphometry statistics.

Cluster-level		Peak-level	Montreal Neurological Institute T1 atlas (mm)			
*p*(false discovery rate corrected)	cluster size	*t* score	*x*	*y*	*z*	Anatomical region
Total						
<a, body mass index>						
0.001	2486	5.4	15.0	−70.5	−58.5	Right cerebellum exterior
		5.0	−7.5	−81.0	−51.0	Left cerebellum exterior
		3.4	6.0	−79.5	−24.0	Right cerebellum exterior
0.005	1719	4.8	−30.0	−93.0	−33.0	Left cerebellum exterior
		4.7	−28.5	−82.5	−54.0	Left cerebellum exterior
		3.9	−31.5	−75.0	−55.5	Left cerebellum exterior
<b, underweight>						
0.000	2522	5.0	0.0	−88.5	6.0	Bilateral cuneus and calcarine cortex
		4.6	−1.5	−73.5	19.5	Left cuneus
		4.4	−10.5	−67.5	43.5	Left precuneus
<c, overweight or obese>						
0.012	1460	4.5	7.5	−79.5	−51.0	Right cerebellum exterior
		4.0	15.0	−67.5	−57.0	Right cerebellum exterior
		3.6	7.5	−78.0	−30.0	Right cerebellum exterior
Men						
<a, underweight>						
0.002	1729	5.3	0.0	−84.0	9.0	Bilateral cuneus and calcarine cortex
		4.6	−1.5	−73.5	16.5	Left cuneus and calcarine cortex
		3.9	0.0	−81.0	25.5	Bilateral cuneus
<b, overweight or obese>						
0.021	1141	4.4	−16.5	−16.5	12.0	Left thalamus proper
		4.3	−10.5	−9.0	13.5	Left thalamus proper
Women						
<a, body mass index>						
0.001	2238	5.8	−30.0	−93.0	−31.5	Left cerebellum exterior
		5.4	−39.0	−88.5	−30.0	Left cerebellum exterior
		4.5	−34.5	−73.5	−60.0	Left cerebellum exterior
0.004	1432	5.0	12.0	−73.5	−58.5	Right cerebellum exterior
		4.1	25.5	−63.0	−60.0	Right cerebellum exterior
		3.9	28.5	−52.5	−60.0	Right cerebellum exterior
<b, overweight or obese>						
0.023	1064	4.4	9.0	−84.0	−51.0	Right cerebellum exterior
<c, obese>						
0.001	1742	3.8	−21.0	−94.5	−30.0	Left cerebellum exterior
		3.7	−45.0	−73.5	−54.0	Left cerebellum exterior
		3.6	−25.5	−87.0	−39.0	Left cerebellum exterior

**FIGURE 5:**
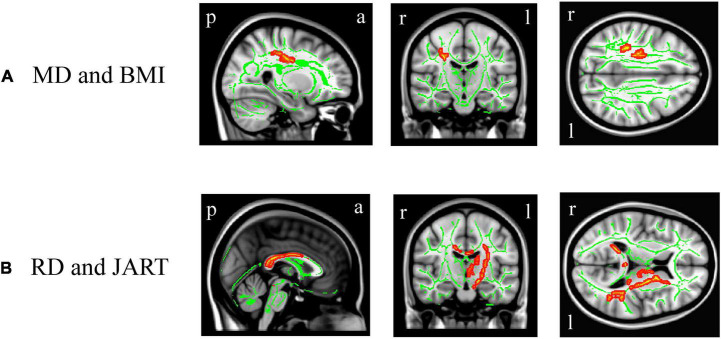
White matter regions where the mean diffusivity (MD) and radial diffusivity (RD) values were correlated with body mass index (BMI) and Japanese Adult Reading Test (JART) score, respectively. BMI was significantly and positively correlated with the right superior longitudinal fasciculus MD value (**A**, *p* < 0.05 corrected), while the JART score significantly and negatively correlated with the corpus callosum and left internal capsule RD value **(B**, *p* < 0.05 corrected). Images coordinate *x* = 27.0 mm, *y* = −17.0 mm, *z* = 34.0 mm **(A)** and *x* = 0.0 mm, *y* = −15.0 mm, *z* = 10.0 mm **(B)**, respectively, in the Montreal Neurological Institute 152 T1 atlas. a, anterior; l, left; p, posterior; r, right.

In DTI, BMI was significantly and positively correlated with the right superior longitudinal fasciculus MD value (Figure 5A, corrected *p* < 0.05), while there was no significant correlation between BMI and the other white matter metrics (FA, AD, or RD). There were no significant differences in the white matter metrics (FA, AD, RD, and MD values) between the underweight and non-underweight groups, the overweight or obese and underweight or normal groups, and the obese and non-obese groups (data not shown). The JART score significantly and negatively correlated with the corpus callosum and left internal capsule RD value (Figure 5B, corrected *p* < 0.05), while there was no significant correlation between the JART score and white matter FA, AD, or MD values.

## Discussion

BMI was negatively correlated with gray matter volumes in the bilateral cerebellum exterior. In group comparisons, gray matter volume in the non-dominant cerebellum exterior were lower in overweight or obese individuals than in underweight or normal individuals. Moreover, gray matter volume in the bilateral cuneus and calcarine cortex, dominant cuneus, and dominant precuneus was lower in underweight individuals than in non-underweight individuals. These findings suggest association of abnormally high or low BMI with reduced gray matter volume in individuals with a wide range of BMI group.

The gray matter regions with reduced volumes in overweight or obese individuals (i.e., cerebellum) are included into those reported in the meta-analyses (Garcia-Garcia et al., 2019; Herrmann et al., 2019). Our sex-related stratification analyses suggested that the bilateral cerebellum exterior volumes reduction is characteristic of women considering that such regional volume reduction in overweight or obese group, obese group, or their correlation with BMI were not observed in men. Alternatively, we have found that the dominant thalamus proper volume reduction was observed only in overweight or obese men. Furthermore, to our knowledge, we have found for the first time that, gray matter volume is reduced in underweight individuals. Our sex-related stratification analyses also suggested that the bilateral cuneus and calcarine cortex, dominant cuneus and calcarine cortex, and bilateral cuneus volume reduction in underweight group is characteristic of men, since such regional volume reduction was not observed in women. Although sex-related regional gray matter (e.g., cerebellum exterior, cuneus, and calcarine cortex) volume reductions were thus observed, sex-specific brain networks/circuits may not be identified in a generalized population, considering a review reporting that sex-related differences of the brain are trivial other than its size (Eliot et al., 2021). This study suggests that individuals who are biased in terms of BMI may show population-specific gray matter volume reductions, considering relatively low rate of overweight (24 among 240 = 10%) or obese (3 among 240 = 1.25%) women and underweight (5 among 142 = 3.5%) men in each sex-group.

An inconsistency was found with our prior study of 294 individuals (Hidese et al., 2018). However, the present findings of reduced gray matter volumes in individuals with higher BMI are more robust, considering that the sample size (*n* = 382) in this study was larger; therefore, the possibility of statistical errors would have been lower than that in the previous (Hidese et al., 2018). Additionally, our inconsistency might have been related to the difference between 1.5 and 3.0 Tesla MRI systems, although the statistical significance was set at the same level. In contrast, a positive correlation between BMI and gray matter volume or increased gray matter volume in obese individuals was not found in our study, which is inconsistent with previous studies (Taki et al., 2008; Yao et al., 2016; Zhang et al., 2017; Huang et al., 2019). Although the details are unexplainable, these inconsistencies might be due to differences in the MRI scanners, parameters, and statistical settings. Our results suggest both higher and lower BMIs are associated with reduced gray matter volume, although the associations will be simplified as merely negative effects of continuous BMI on regional gray matter volume in VBM correlational analyses or will be changed to even no significant correlation between continuous BMI and total gray matter volume.

Our DTI results are inconsistent with those of correlational studies in 56 individuals (Xu et al., 2013), 33 individuals (Kullmann et al., 2016), two independent cohorts (*n* = 364 and 1064; Repple et al., 2018), and 1255 individuals (Zhang R. et al., 2018) as well as with the association between obesity and white matter microstructures noted in studies of 103 (Stanek et al., 2011), 46 (van Bloemendaal et al., 2016), and 50 (Chen et al., 2018) individuals. Except for that in two studies (Repple et al., 2018; Zhang R. et al., 2018), the number of participants (*n* = 382) was larger than that in many previous DTI studies (Stanek et al., 2011; Xu et al., 2013; Kullmann et al., 2016; van Bloemendaal et al., 2016; Chen et al., 2018), which would have helped prevent statistical (type 2) errors. Moreover, no BMI-related association and that of its classifications are consistent with the results of our previous study (Hidese et al., 2018) in terms of white matter region, which support that the effects of BMI will not be represented on white matter microstructures, differing from patients with major depressive disorder. Although BMI was not associated with white matter microstructures, BMI was positively correlated with TICV, especially total white matter volume. To our knowledge, such a correlation has not been previously reported. Considering that lipids are the main component of myelin in the white matter region (Nave and Werner, 2014; Sharma et al., 2020), metabolic bodies may be associated with abnormal lipid accumulation, which results in an increase in total white matter volume and possibly its concomitant decrease in total CSF volume.

The JART score was lower in overweight or obese individuals than in underweight or normal individuals. However, the JART score was not correlated with continuous BMI (*r* = −0.06, *p* = 0.26), and rather higher in obese individuals than in non-obese individuals although the latter finding would not be conclusive owing to the very small sample size (*n* = 6). A large (*n* = 17419) longitudinal study (Kanazawa, 2013) reported that lower childhood IQ was associated with higher BMI (being overweight or obese) in adulthood even when educational attainment, which is a proxy for socioeconomic status (Bambra et al., 2012; Bambra et al., 2013; Cohen et al., 2013; Li et al., 2019), is controlled for. Inconsistent with the previous longitudinal study (Kanazawa, 2013), our data showing no correlation between JART score and continuous BMI would not eventually support that lower IQ leads to excessive caloric intake and increased BMI.

Regarding the underlying pathological mechanism, our data suggest that systemic inflammation (Beyer et al., 2019) and adiposity (Willette and Kapogiannis, 2015) in overweight or obese individuals may be related to gray matter volume reduction in the normal-aged (46.9 ± 14.3 years) population. The present study suggests that regional gray matter volume reduction according to abnormal BMI increase is likely to appear in women although the proportion of men (*n* = 35) was higher among being overweight or obese individuals (*n* = 62). Malnutrition in underweight individuals may be related with the progression of gray matter volume reduction (Bourre, 2006b,a). Although this study analyzed MRI images in a population without psychiatric disorders, patients with anorexia nervosa have also been reported to show gray matter volume reductions (Seitz et al., 2014; Zhang S. et al., 2018). Although young women are more likely to lose body weight in Japan (Day et al., 2004; Kagawa et al., 2007) and the proportion of women (*n* = 25) was indeed higher among underweight individuals (*n* = 30) in this study, the presented risk of being underweight is suggested to appear rather in men since regional gray matter volume reduction was not seen in underweight women. Our data suggest that a balanced BMI (i.e., being normal) is beneficial for the structural condition of the gray matter region, even in individuals without psychiatric disorders.

This study has several limitations. First, sex-related differences were found in BMI, although the effects of sex were controlled for in the ANCOVA and MRI analyses in total participants. In addition, sex-related stratification analyses were performed for VBM results to investigate the effects of sex-related differences specifically. Second, the number of obese individuals was small (*n* = 6), suggesting that the statistical power of the results was less than that of underweight (*n* = 30) and overweight (*n* = 56) individuals. This lack of statistical power may result in no significant difference in gray and white matter metrics in the obese group compared with the non-obese group. To support association between unusual high or low BMI and gray matter volume decreases and draw any conclusion about the obese group, studies in much more large number of obese individuals are required. Third, this BMI study has not included data of body composition profiles (Muller et al., 2016) measured by anthropometric indicators of body fat (Sommer et al., 2020) or Dual-energy X-ray Absorptiometry (Johnson Stoklossa et al., 2016; Borga et al., 2018; Messina et al., 2020) and biomarkers of inflammation (Aleksandrova et al., 2021; Hart et al., 2021; Hidese et al., 2021). Fourth, to avoid type 2 errors, we chose to use a peak-level of *p* < 0.001 (uncorrected) and a cluster-level of *p* < 0.05 (FDR corrected), but not to use more conservative correction of both peak-level and cluster-level of *p* < 0.05 (FDR corrected) for multiple comparisons in VBM. Vice versa, our VBM analyses may include any type 1 errors although the statistical significance was intended to set at the same level as that in our prior study (Hidese et al., 2018). Finally, although BMI changes were associated with regional gray matter reduction, the time course could not be elucidated in this cross-sectional study. Further longitudinal MRI studies to investigate the association of BMI, body composition profiles, and biomarkers of inflammation with brain structure are warranted.

In conclusion, BMI was negatively correlated with regional gray matter volume. In group comparisons, regional gray matter volumes were lower in overweight or obese individuals than in underweight or normal individuals and in underweight individuals than in non-underweight individuals. Moreover, the association of BMI and its classifications with regional gray matter volume reductions showed sex-related differences. These results suggest that unusual high or low BMI is associated with regional gray matter volume reductions. Thus, body weight control is suggested to be beneficial for a healthy brain.

## Data availability statement

The original contributions presented in this study are included in the article/supplementary material, further inquiries can be directed to the corresponding author.

## Ethics statement

The studies involving human participants were reviewed and approved by the Ethics Committee of the National Center of Neurology and Psychiatry. The patients/participants provided their written informed consent to participate in this study.

## Author contributions

SH and HK designed and supervised the study, respectively. SH, MO, and YHY made the diagnoses. JM, II, YY, and KH recruited and interviewed the participants. MO and YHY were collected the MRI data and analyzed by SH who performed the statistical analyses and wrote the manuscript, which was approved by all authors.

## Footnotes

1
http://www.neuro.uni-jena.de/cat/

2
http://www.fil.ion.ucl.ac.uk/spm

## Abbreviations

AD: axial diffusivity
ANCOVA: analysis of covariance
ANOVA: analysis of variance
BMI: body mass index
CAT: Computational Anatomy Toolbox
CSF: cerebrospinal fluid
DTI: diffusion tensor imaging
FA: fractional anisotropy
FDR: false discovery rate
IQ: intelligence quotient
JART: Japanese Adult Reading Test
MD: mean diffusivity
MRI: magnetic resonance imaging
NCNP: National Center of Neurology and Psychiatry
RD: radial diffusivity
SPM: statistical parametric mapping
TICV: total intracranial volume
VBM: voxel-based morphometry.
